# Impact of Multifunctional Adjuvants on Efficacy of Sulfonylurea Herbicide Applied in Maize (*Zea mays* L.)

**DOI:** 10.3390/plants12051118

**Published:** 2023-03-02

**Authors:** Robert Idziak, Angelika Sobczak, Hubert Waligora, Piotr Szulc

**Affiliations:** 1 Department of Agronomy, Poznan University of Life Sciences, Dojazd 11, 60-632 Poznan, Poland; 2 Research and Education Center Gorzyń, Wojska Polskiego 28, 60-637 Poznan, Poland

**Keywords:** multifunctional adjuvants, maize, nicosulfuron, weed control

## Abstract

To reduce the cost of intensive herbicide application and environment pollution and enhance biological effectiveness, effective multifunction adjuvants should be used. A field study was conducted in 2017–2019 in midwestern Poland in order to assess the effects of new adjuvant formulations on the activity of herbicides. Treatments included the herbicide nicosulfuron at recommended (40 g ha^−1^) and reduced rates (28 g ha^−1^) alone and with the addition of tested MSO 1, MSO 2, and MSO 3 (differing in the type and amount of surfactants), as well as standard (MSO 4 and NIS) adjuvants. Nicosulfuron was applied once during the 3–5 leaf stage of maize. Results indicate that nicosulfuron with the tested adjuvants provided satisfactory weed control equivalent to that provided by standard MSO 4 and better than that provided by NIS. Nicosulfuron applied with the tested adjuvants led to a similar grain yield of maize as that achieved with standard adjuvant treatments and much higher than that measured in untreated crops.

## 1. Introduction

Maize is characterized by poor competitiveness in relation to weeds [1,2], and its late sowing in spring makes weed control the most important treatment for this species. The presence of weeds in the field results in yield losses of 13–30% and, in extreme cases, of strong weed pressure exceeding a 70% reduction in grain yield [3,4]. Maize shows a negative response to weed pressure, especially in the first weeks after sowing [5]. Leaving a small number of, among others, *Chenopodium album* and *Echinochloa crus-galli* in the field, along with unfavorable weather conditions, is one of the most significant factors limiting maize yields and income from maize cultivation [6]. Researchers [7] indicated that the correct selection of an active substance appropriate to the community species composition and, preferably, mixtures of substances with different mechanisms of action, can guarantee the effective elimination of weeds from the plantation.

Weed control in arable crops is mainly carried out using chemical control measures, although the emphasis is primarily on the use of non-chemical methods and, as a last resort, herbicides [8]. Increasing requirements are placed on plant protection products in terms of their effectiveness, cost of application [9], and safety for the crop and people performing the treatment. It therefore becomes important to properly select the herbicide according to the composition of the weed community, dose, application date, and favorable weather conditions [10], i.e., appropriate temperature and humidity for at least several hours a day during the several days after the treatment. Depending on the active ingredient, herbicides differ in terms of requirements for the minimum temperature necessary for their effective action [11]. The final effect of the treatment also depends on weed sensitivity, which is also associated with weather conditions [12]; in most cases, the optimum temperature for herbicides ranges between 10–20 and 25 °C [13]. Moderate temperature and high air humidity enhance the effect of herbicides [14] due to better moistening of the leaf surface [15], higher retention [16], and reduced evaporation of spray droplets [17].

Chemical crop protection against pests is an important part of agricultural practice, also having an impact on the environment; therefore, it is advisable to use solutions that allow for maximum effectiveness with the least possible environmental impact [18]. Improper herbicide selection and treatment errors translate into low herbicide efficacy or the emergence of secondary weeds in the second half of the growing season, resulting in a decrease in crop yield and quality [19]. Under field conditions, only a small proportion of the herbicide reaches the site of action, and treatment effectiveness is determined by only one-tenth of the applied herbicide dose [20]. From the point of view of the user of plant protection products and the environment, it is justified to improve this element of agricultural technology to minimize the losses of the active substance so that the lowest possible dose of the product can be applied, with the highest possible proportion reaching the site of action in the plant.

Pesticides must be used according to the principles of integrated pest management, i.e., non-chemical methods should be used first and herbicides as a last resort. Changes in the law allow farmers to reduce the doses of chemicals, provided that they are correctly applied, in accordance with the principles of good plant protection practices. In order to maintain high effectiveness of reduced herbicide doses, it is advisable to add substances supporting their action to the spray liquid [21]. Adjuvants that directly or indirectly affect the action of the active ingredient of herbicides are classified as activating adjuvants, and those that alter the performance of the product formulation and spray liquid are classified as modifying adjuvants [22]. In terms of chemical structure, activating adjuvants are divided into surfactants, oils, inorganic salts, or fertilizers, and multicomponent (multifunctional) adjuvants are based on a mixture of two or more compounds [23].

The effectiveness of herbicides is influenced by a number of factors, including the selection of the agent, its dose, date and method of application, weather conditions during and after the application [13], the weed species and their morphological and anatomical properties, and the course of physiological and biochemical processes occurring in them [24]. Knowledge of these processes is necessary to develop recommendations and plant protection programs that enable effective and economical weed control. An equally important aspect is to limit the negative impact of plant protection products on the environment, protected crops [25], and the quality of the harvested plant material [26].

On this route from the sprayer to the site of action, herbicides encounter many obstacles, which adjuvants help to overcome. The beneficial effect of adjuvants depends on their type. Adjuvants based on a single chemical substance are not able to reduce the adverse impact of many factors of a very diverse nature; therefore, it is necessary to use multicomponent adjuvants with multidirectional action [27]. Adjuvants added to the spray tank help herbicides overcome barriers that restrict their access to the leaf surface and plant interior. However, achieving a positive effect of herbicide application requires the selection of a suitable adjuvant adapted to the specific herbicide group and even the active ingredient, as an incorrectly selected adjuvant may even reduce the herbicide’s performance [28].

The improved performance of herbicides applied with adjuvants is primarily the result of increased herbicide uptake [29]. However, the effect of adjuvants, especially multicomponent adjuvants, is multifaceted and can occur at the time of preparation of the spray liquid in the spray tank, at the moment of contact and retention of droplets on the leaf surface, or during their absorption and translocation to the site of action in the plant [30]. Thanks to the surfactants in their formulations, adjuvants increase the retention of spray liquid droplets and contribute to a more uniform coverage of the plant surface by reducing the surface tension and the angle of contact of spray liquid droplets on the surface of weed leaves [31]. In turn, the presence of penetrants, which include vegetable oils, increase the penetration of herbicides through cuticular wax and cell walls, as well as stomata [32]. 

Single-component adjuvants do not allow for full control of all the factors that adversely affect the action of herbicides in maize and therefore do not guarantee a satisfactory level of protection against weeds. In order to achieve high treatment efficiencies at reduced costs and limited environmental impact, it is essential to add effective, herbicide-specific adjuvants with a multidirectional effect, which make it possible to reduce the herbicide dose while maintaining high efficacy. The reduction in treatment costs and impact on the habitat is possible with the tested adjuvants because they are cheaper to produce, compatible, non-crystallizing, and safer for the environment. Increased safety results from the fact that the substances included in the formulation of the tested adjuvants were also analyzed for their impact on the environment.

The aim of this study was to evaluate the effect of the newly developed formulations of multifunctional adjuvants on the physicochemical properties of the spray liquid containing nicosulfuron, their impact on the efficacy of herbicides applied at reduced rates, and phytotoxicity to plants, as well as maize grain yield and its parameters.

## 2. Results

Weed flora of experimental fields consisted of over a dozen species of broad-leaved weeds and grasses. The major broad-leaved weeds were lambsquarters (*Chenopodium album* L.), small-flowered crane’s-bill (*Geranium pusillum* L.), black-bindweed (*Fallopia convolvulus* (L.) Á.), and field pansy (*Viola arvensis* L.). Among the grassy weeds, common barnyardgrass (*Echinochloa crus-galli* (L.) P. Beauv) was included. The total weed fresh weight varied over the years from 2313 to 5663 g m^−2^ (Table 1, Table 2 and Table 3). Data included all weed species turned out during the study, such as *Anthemis arvensis* L., *Centaurea cyanus* L., *Galium aparine* L., *Tripleurospermum maritimum* (L.) W.D.J.Koch, *Polygonum aviculare* L., *Anchusa arvensis* (L.) M.Bieb., *Veronica hederifolia* L., *Amaranthus retroflexus* L., *Cirsium arvense* (L.) Scop., *Fumaria officinalis* L., *Lamium amplexicaule* L., *Stellaria media* (L.) Vill., *Plantago major* L., *Galinsoga parviflora* Cav., *Capsella bursa-pastoris* (L.) Medik., and *Thlaspi arvense* L. (Table 1).

**Figure 1:**
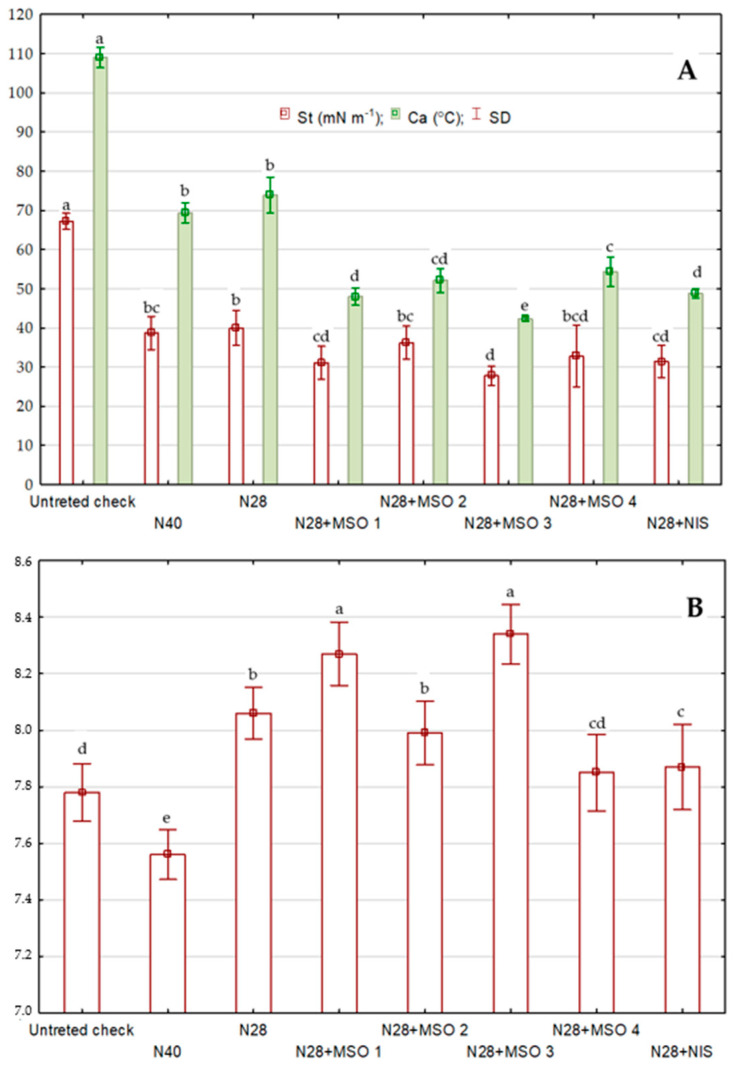
Impact of adjuvants (MSO 1, 2, 3, and 4 and NIS) on surface tension (St), contact angle (Ca) (**A**), and reaction of spray liquid (**B**) with nicosulfuron (N). Means followed by the same letter in the column do not differ according to Tukey’s test at *p* = 0.05. SD—standard deviation.

Spray liquid pH range from 7.56 to 8.27 compared to 7.78 in the untreated check (water). Tested adjuvants MSO 1 and MSO 3 raised the pH of spray liquid more than MSO 2 and standard adjuvants (Figure 1). Results indicate that nicosulfuron (N) reduced St and Ca compared to the untreated check. The addition of MSO 1 and MSO 3 to nicosulfuron applied at a lower rate reduced St to 31 and 28 mN m^−1^, respectively, the addition of MSO 2 reduced St to 36 mN m^−1^ compared to 31–33 mN m^−1^ with standard adjuvants. Similar relationships were found for Ca.

In 2017, nicosulfuron at recommended and reduced rates effectively controlled only ECHCG (Table 2). WCE (weed control efficacy) of other weeds was mostly lower when nicosulfuron was applied at a reduced rate and higher when applied with MSO 1 and MSO 3 at similar or higher levels than standard adjuvants. The total WCE of nicosulfuron with MSO 3 (84%) was higher than that with MSO 1 (68%) and standard adjuvants MSO 4 and NIS (63 and 71%). In 2018, nicosulfuron considerably controlled GER and VIO (100%), independently of rates and adjuvants. CHE, ECH, and FAL control was significantly improved by the application of nicosulfuron at a reduced rate with all tested adjuvants, similar to standard adjuvant MSO 4. Generally, nicosulfuron applied with adjuvants improved total weed control with MSO 3 and MSO 1, much better when NIS, and slightly better with MSO 4. In 2018, nicosulfuron considerably controlled GER and VIO (100%), independently of rates and adjuvants. CHE, ECH, and FAL control was significantly improved by the application of nicosulfuron at a reduced rate with all tested adjuvants, similar to standard adjuvant MSO 4. Generally, nicosulfuron applied with adjuvants improved total weed control with MSO 3 and MSO 1, much better with NIS, and slightly better with MSO 4. The 2019 results confirm that the application of a lower herbicide rate decreased efficacy. The addition of tested adjuvants MSO 1, MSO 2, and MSO 3 with a lower rate of nicosulfuron improved control of CHE from 44–79 (N without adjuvants) to 95%, 95–99% ECH, 85–91% FAL, and 100% VIO. Nicosulfuron applied alone or with tested adjuvants controlled GER only to 70–77 and 74–79%. Total WCE of N28 applied with adjuvants MSO 1, 2, 3, and 4 did not vary (93–94%). Generally, total WCE indicated that adjuvants MSO 1–3 work as well as standard MSO 4 and much better than standard adjuvant NIS. 

Tested herbicide treatments, regardless of rates and adjuvants, were safe for maize plants; no injury was noted in any year (Table 3). Weed control using nicosulfuron (N) applied alone or with adjuvants had an effect on maize grain yield. The lowest grain yield in each year of the study was obtained from the control object. The application of N at full dose resulted in a significant increase in grain yield. In 2017, the inclusion of test and standard adjuvants in the spray liquid containing N28 increased grain yield to 17.1 with MSO 1, 18.7 t ha^−1^ with MSO 3, 17.3 with MSO 4, and 17.9 t ha^−1^ with NIS; however, these differences were not statistically significant. In the second and third years of the study, the yield of maize grain from the objects treated with N40 was 7.2 and 9.0 t ha^−1^, respectively. Lowering the N dose resulted in a decrease in the yield to 6.7 and 6.3 t ha^−1^ compared to 2.8 and 2.2 t ha^−1^ in the control object, respectively. The incorporation of the experimental adjuvants MSO 1, MSO 2, and MSO 3 into the liquid resulted in an increase in grain yield to 9.4 and 9.0, 8.2 and 9.0 and 7.9 and 10.2 t ha^−1^, respectively. The highest yield in 2018 was obtained from the N28 + MSO 1 object, and in 2019, from the N28 + MSO 3 object, although these differences were not statistically confirmed. The thousand-kernel weight (TKW) of maize from the control object in the first year of the research was 277 g and 294 g from the object in which N40 was applied (Table 4). The application of N28 without adjuvants resulted in a statistically significant reduction in TKW to 267 g. TKW from the objects in which N28 was applied with adjuvants MSO 1 and MSO 3 and the adjuvants MSO 4 and NIS was 297 and 305 g and 300 and 297 g, respectively; statistical analysis showed no differences between these objects. In 2018 and 2019, the lowest TKW was found in the control objects (244 and 259 g, respectively). Control of weeds with herbicide N, regardless of the dose and the absence or presence of adjuvants, resulted in an increase in TKW, with no significant differences between the research objects, although there was a trend indicating higher TKW in objects with N28 and adjuvants. 

Spray liquid parameters had both a negative direct effect and a positive correlation coefficient (Table 4). Spray liquid pH had a positive significant and was considerably correlated with herbicide efficacy, except treatment N28 + MSO 3; Ca with N28 + MSO 1, MSO 2, MSO 3, and NIS, St with N28 + MSO 1, MSO 2, and MSO 4; and NIS. The effect of Ca on efficacy was negative only with N28. The effect of spray liquid on grain maize yield had a mostly negative correlation, which was significant with N40, N28 + MSO 4, N28 + NIS (Ca), N28 + MSO 1 (St), and N28 + MSO 2 (pH).

## 3. Discussion

The composition of weed communities underwent slight changes, and in the three-year period, the presence of 21 weed taxa was found, including the strongest competition with crop plants *Ch. album*, *E. crus-galli, G. pusillum, F. convolvulus*, and *V. arvensis*, belonging to *Polygono-Chenopodietalia*, as well as *Echinochloa-Setarietum* and *Polygono-Chenopodietalia* weed communities in maize [33,34]. Knowledge about weed community structure is critical to plan an effective weed management system over time [35]. 

Air humidity during herbicide treatments in Research and Education Center (REC) Brody was 60–70% in individual years at temperatures above 13 °C. Nicosulfuron belongs to the sulfonylurea group, and according to [36], herbicides from that group control weeds more effectively when the day and night temperature is 25/23 °C and atmospheric humidity is closer to 90–100% than 40–50% [37]. Considering the weather conditions during and after the treatments, it can be concluded that that herbicides were applied under favorable conditions.

The results of numerous studies have indicated that properly selected adjuvants, especially multicomponent adjuvants, have a greater impact on increasing the effectiveness of herbicides [38]. The results of the present study confirm that the tested multicomponent adjuvants significantly improved the effectiveness of herbicides applied in maize. It should be noted that the beneficial effect of these adjuvants was comparable and often better than that of the standard adjuvant containing methyl esters, surfactant, and pH buffer. The results also indicate a significantly better interaction of herbicides with the adjuvants MSO 1, MSO 3, and MSO 2 and the standard adjuvant MSO 4 than with the simple adjuvant classified as a surfactant (NIS).

Minimizing the risk of spray droplet drift and improving the retention and absorption of herbicide active substances is possible by reducing the surface tension and the angle of contact between the droplets and weed leaf surfaces [39]. The angle of contact between liquid droplets and the solid surface decreases with decreasing tension, but, ultimately, it also depends on the characteristics of the surface on which the droplet has settled. Lower contact angle values indicate the tendency of the liquid to spread and adhere to the surface, while high values indicate the tendency of the surface to repel water droplets [40]. The results of numerous studies have indicated that the surface tension of most mixtures of plant protection products ranges from about 30 to 73 mN m^−1^ [41,42]. Surface tension is obviously one of many factors determining the effect of herbicides; therefore, even when the value of this parameter is within the optimal limits (30–40 mN m^−1^), it does not necessarily translate into high herbicide effectiveness. The study results indicate that the surface tension of nicosulfuron-containing spray droplets was moderate (38–40 mN m^−1^), regardless of the dose of the agent. The adjuvants under study further reduced the surface tension to a lower or higher extent, depending on the formulation of the agent, to values within the optimal range (30–40 mN m^−1^).

The pH of the spray liquid affects the solubility of herbicides and their hydrolysis [43]. The efficacy of nicosulfuron depends on the pH of the spray liquid and the adjuvant added to the mixture [44]. At higher pH values above 7, the solubility of sulfonylurea herbicides increases, which usually enhances their activity. Low solubility of sulfonylureas can be expected when the pH of the spray liquid is lower than the pKa of the active substances—for nicosulfuron, 4.3–4.6 [45]. The best adjuvants in such situations are those that contain a surfactant or oil and a buffering substance that raises the pH of the spray liquid. The results of the current study indicate a beneficial effect of the tested adjuvants, i.e., MSO 3 and MSO 1, on nicosulfuron action. Positive correlation of herbicide efficacy with spray liquid properties, especially St and Ca of droplets, mostly depends on the adjuvants [46]. Our results indicate that efficacy was correlated with both St and Ca but also with the pH of the spray liquid. Positive correlations of the efficacy of sulfonylurea herbicides with pH are widely reported [47].

Achieving the expected increase in yield requires, among other things, a significant reduction in weed competition in maize cultivation. Under REC Brody conditions, weed species reduced maize grain yield in 2017, 2018, and 2019 by 33–54%, 58–70%, and 65–78%, respectively (untreated control vs. treatments). Thus, the use of herbicides allows enables attainment of at least 33% higher grain yield, with a close to 90% increase in yields in fields where weeds were not controlled [48]. 

High efficiency of treatments is achieved using full doses of herbicides recommended by the manufacturer, although the same effect can be obtained using reduced doses of herbicides, especially under favorable conditions. However, it is recommended to add adjuvants to the spray liquid, primarily multicomponent, multifunctional adjuvants with a broad, comprehensive effect [49]. It should be noted that the beneficial effect of adjuvant additives is obtained only when the controlled weeds are sensitive to a given active ingredient, e.g., effective *Echinochloa crus-galli* elimination from a maize field is primarily the result of high sensitivity to nicosulfuron [38] and appropriately selected adjuvants [50]. The new formulations of the experimental adjuvants influenced the performance of nicosulfuron to the same extent and often even more effectively than the multicomponent standard adjuvant and markedly better than the adjuvant containing only the surfactant. Multidirectional adjuvants appropriately selected for a given herbicide allows for a reduction in the dose of the agent while maintaining its high effectiveness.

## 4. Materials and Methods

Field studies were conducted in 2017–2019 in Research and Education Center Brody (REC Brody, N 52° 43′ 07″ E 016° 17′ 30″), belonging to Poznan University of Life Sciences, central–west Poland, to evaluate the effects of experimental adjuvants on the efficacy of herbicides applied at reduced rates for weed control management in maize compared to standards adjuvants. The experiment was arranged in a randomized complete block design in 5 (adjuvants) and 1 herbicide arrangements with 4 replications. Each plot contained 4 rows spaced 70 cm apart with a plot length of 9 m; the total plot area was 25.5 m^−2^. The soil was classified as a loamy sand with a clay content of 16%, 58% sand, and 26% silt in years with pH of 6.6, 5.9, 6.3, and 1.2–1.4% organic matter. White lupin, winter wheat, and winter barley were planted in the following years. Maize cultivar PR39H32 was sown using a single-row Monosem driller to a depth of 4 cm on 6 May 2017, 25 April 2018, and 24 April 2019 at 80,000 seeds ha^−1^. The plants were harvested from 2 middle rows of each plot on 29, 4, and 10 September. Nicosulfuron (N, Nisshin 040 SC, nicosulfuron 40 g L^−1^, ISK Bioscences, Brussels, Belgium) was used at rate recommended by the manufactures (40 g L^−1^) and a reduced rate (28 g L^−1^). Three experimental and two standard adjuvants were used. Herbicides were used at 14–15 BBCH of maize without and with the addition of tested new formulations of adjuvants MSO 1 and MSO 2 (Agromix Ltd., Niepolomice, Poland) introduced for research in 2018 (adjuvants differ in the type and amount of surfactants, and MSO 3 (Agromix Ltd., Niepolomice, Poland) at 1.5 L ha^−1^. Depending on their chemical composition, their function was, among others, to ensure maximum solubility of the herbicide in the spray liquid, to prevent crystallization of the herbicide on the surface of weed leaves, to reduce evaporation and drift of spray droplets, to protect the herbicide from being washed away by rain, and to ensure rapid and increased movement of s.a. deep into plant tissues and cells. They were expected to improve herbicide efficacy regardless of conditions during and after treatments. Standard adjuvants were used as surfactants (NIS, Trend 90 EC, ethoxylated isodecyl alcohol, DuPont, Paris, France) at 0.1%, and methylated esters (MSO 4, Atpolan Bio 80 EC, Agromix, Niepolomice, Poland) at 1.5 L ha^−1^ (Table 5).

Adjuvant and herbicide treatments were applied with a wheelbarrow CO_2_-pressurized sprayer equipped with TeeJet XR 11015 VS flat fan nozzle tips (50 cm spacing) with a boom height of 50 cm calibrated to deliver 230 L ha^−1^ at a pressure of 0.22 MPa and a ground speed of 3.5 km h^−1^. Weeds were collected 42 days after application from each plot, from randomly selected areas (2 × 0.35 m^−2^), and the efficacy of treatments was determined on the basis of fresh mass reduction from the treated plots compared to the untreated check. Weed control efficacy (WCE) was calculated according to the following formula [51]: WCE = [(W_c_ − W_t_)/W_c_] × 100, where W_c_ is the weed fresh weight in the control, and W_t_ is the weed fresh weight in the treated plot. Maize injuries a scale ranging from 0 (no injury) to 100% (complete plant death) were assessed 14, 28, and 42 days after application. Because no injuries were observed, data were are reported as yearly averages.

The surface tension (St) and contact angle (Ca) were measured 0.1 s after contact of spray liquid droplet with the surface and estimated by a KSV Optical tensiometer, model Theta Lite (KSV Instruments Ltd., Helsinki, Finland), equipped with a camera taking over 60 photos per second (frame interval: 16 ms) designed for the measurement of physical properties of liquids. The pH spray liquids were measured using an Elmetron pH conductometer CPC-505 equipped with an EPS-1 electrode. Physicochemical measurements were performed at a constant room temperature of 20 ± 1 °C and relative humidity of 55–60%. Analyses were performed in the first and second year of the study and pooled across years. 

To evaluate the effect of the tested MSO 1, MSO 2, and MSO 3 adjuvants on the physicochemical properties of spray liquid with nicosulfuron, its efficacy and impact on grain yield statistical procedures were determined using Statistica 13 software (StatSoft Inc., Tulsa, OK, USA). Data were subjected to ANOVA (analysis of variance), and protected Tukey’s HSD was used to separate treatment means at *p* = 0.05. Percent ratings of weed control were arc-sine transformed prior to analysis to correct for unequal variance. Data in tables are reported as non-transformed. Year-over-year treatment interactions were not significant; therefore, yearly data are presented separately. To measure a relationship between variables St—efficacy, St—yield, Ca—efficacy, Ca—yield, pH—efficacy, and pH—yield for each treatment, correlation coefficients (r) were estimated using Statistica to study positively and negatively correlated characters with physical properties of spray liquid and efficacy of herbicide and grain maize yield. 

In all years of the study, weather conditions at the time of herbicide application were rather favorable for their efficacy. Meteorological data from application time and 7 days after are shown in Table 6. 

## Figures and Tables

**Table 1:** Phytosociological classification and abundance of species in the community.

Species	Years of Studies			Life Cycle
	2017	2018	2019	
	g m^−2^			
Characteristic species for *Polygono-Chenopodion* alliance				
*Fumaria officinalis*	-	-	36	annual
Characteristic species for *Aperion spicae-venti* alliance				
*Veronica hederifolia*	-	11	29	annual
Characteristic species for *Polygono-Chenopodietalia* order				
*Chenopodium album*	4370	1261	1013	annual
*Capsella bursa-pastoris*	35	64	-	annual
*Echinochloa crus-galli*	165	270	132	annual
*Geranium pusillum*	515	22	539	annual
Characteristic species for *Centauretalia cyani* order				
*Centaurea cyanus*	32	-	59	annual
*Anthemis arvensis*	17	22	-	annual
Characteristic species for *Plantaginetalia majoris* order				
*Plantago major*		10		perennial
Characteristic species for *Stellarietea mediae* class				
*Viola arvensis*	76	15	8	annual
*Anchusa arvensis*	-	74	41	annual
*Stellaria media*	-	31	62	annual
*Polygonum aviculare*	-	30	51	annual
*Tripleurospermum maritima* ssp. *inodora*	23	32	-	biennial
*Thlaspi arvense*	71	29	-	annual
Characteristic species for *Artemisietea vulgaris* class				
*Cirsium arvense*	36	-	47	perennial
*Gallium aparine*	18	16	-	annual
Characteristic species for *Lamio-Veronicetum* association				
*Lamium amplexicaule*	-	15	31	annual
Characteristic species for *Galinsoga-Setarietum* association				
*Galinsoga parviflora*	4	-	29	annual
Accompanying species				
*Fallopia convolvulus*	301	539	194	annual
*Amaranthus retroflexus*	-	20	42	annual

**Table 2:** Impact of adjuvants on WCE in 2017–2019.

Treatment	CHE			ECH			GER			FAL			VIO			Total		
	I	II	III	I	II	III	I	II	III	I	II	III	I	II	III	I	II	III
Untreated check g m^−2^	4370	1261	1013	165	270	132	515	22	539	301	539	194	76	15	8	5663	2461	2313
**Weed Control Efficacy %**																		
N40	60 c^4^	62 b	79 b	99 a	94 a	95 ab	72 ab	100 a	77 a	79 a	75 a	76 b	75 ab	100 a	67 cd	60 b	67 cd	81 b
N28	45 d	45 c	44 c	97 a	90 ab	90 b	56 c	100 a	70 a	70 a	52 b	62 c	56 c	100 a	56 d	48 b	56 d	52 c
N28																		
+ MSO 1	65 bc	88 a	95 a	100 a	98 a	99 a	71 ab	100 a	79 a	76 a	72 a	91 a	68 ab	100 a	88 ab	68 ab	88 ab	94 a
+ MSO 2	-	83 a	95 a	-	90 ab	95 ab	-	100 a	79 a	-	74 a	89 a	-	100 a	82 ab	-	82 ab	94 a
+ MSO 3	90 a	91 a	95 a	100 a	98 a	95 ab	80 a	100 a	74 a	76 a	73 a	90 a	84 a	100 a	90 a	84 a	90 a	94 a
+ MSO 4	77 b	82 a	9 a	100 a	92 ab	96 ab	67 abc	100 a	78 a	80 a	70 a	85 a	71 ab	100 a	84 ab	71 ab	84 ab	93 a
+ NIS	69 bc	71 ab	78 b	100 a	83 b	97 a	61 bc	100 a	61 b	74 a	69 a	76 b	63 ab	100 a	74 bc	63 ab	74 bc	81 b

I, II, and III—2017, 2018, and 2019, respectively; N40—nicosulfuron at 40 g ha^−1^; N28—nicosulfuron at 28 g ha^−1^; ^1^ MSO 1, 2, 3, and 4 at 1.5 L ha^−1^; NIS at 0.01%; ^2^ MSO 2 included in the study from 2018; ^3^ CHE: *Chenopodium album*; ECH: *Echinochloa crus-galli*; GER: *Geranium pusillum*; FAL: *Fallopia convolvulus*; VIO: *Viola arvensis*. ^4^ Including all weed species turned out during the study. Means followed by the same letter in the column do not differ according to Tukey’s test HSD at =0.05.

**Table 3:** Influence of adjuvants on grain yield of maize.

Adjuvant	Herbicide g ha^−1^	Phytotoxicity %	Grain Yield T ha^−1^			TKW ^3^ g		
		2017–2019	2017	2018	2019	2017	2018	2019
Untreated check		0	8.6 c	2.8 c	2.2 c	277 ab	244 b	259 b
N40	40	0	16.5 a	7.1 ab	9.0 ab	295 a	271 a	296 a
N28	28	0	13.0 b	6.7 b	6.3 b	267 b	275 a	291 a
N28								
+ MSO 1 ^1^	28	0	17.1 a	9.4 a	9.0 ab	297 a	292 a	300 a
+ MSO 2 ^2^	28	0	-	8.3 ab	9.0 ab	-	288 a	313 a
+ MSO 3	28	0	18.7 a	7.9 ab	10.2 a	305 a	285 a	316 a
+ MSO 4	28	0	17.9 a	8.1 ab	9.3 ab	300 a	275 a	316 a
+ NIS	28	0	17.3 a	7.4 ab	8.0 ab	297 a	285 a	287 a

^1^ MSO 1, 2, 3, and 4 at 1.5 L ha^−1^; NIS at 0.01%; ^2^ MSO 2 included in the study from 2018; ^3^ TKW: 1000 kernel weight. Means followed by the same letter in the column do not differ according to Tukey’s test HSD at =0.05.

**Table 4:** Correlation coefficients among spray liquid properties, herbicide efficacy, and grain yield of maize.

Treatment	Parameter	Efficacy	Yield
		r	
N40	St	0.2750	−0.5049
	Ca	0.4389	−0.6106 *
	pH	0.7043 *	−0.5724
N28	St	−0.5640	0.3566
	Ca	−0.6748 *	0.3897
	pH	0.7109 *	−0.5735
N28 + MSO 1	St	0.6999 *	−0.6912 *
	Ca	0.7013 *	−0.3409
	pH	0.7241 *	−0.5705
N28 + MSO 2	St	0.8319 *	−0.3958
	Ca	0.7287 *	−0.5319
	pH	0.7281 *	−0.5819 *
N28 + MSO 3	St	0.1259	0.1501
	Ca	0.6733 *	−0.5592
	pH	−0.1282	0.1605
N28 + MSO 4	St	0.7323 *	−0.2508
	Ca	0.4536	−0.6380 *
	pH	0.7262 *	−0.5654
N28 + NIS	St	0.7174 *	−0.5336
	Ca	0.6796 *	−0.5963 *
	pH	0.7007 *	−0.5709

St—surface tension; Ca—contact angle; r—correlation coefficient; * the indicated correlation coefficients are significant at *p* ≤ 0.05.

**Table 5:** Tested and standard adjuvants applied in the field experiments in 2017–2019.

Abbreviation	Composition	Rate per ha
MSO 1	Methyl esters of rapeseed oil fatty acids, surfactants, and pH buffering spray liquid	1.5 l
MSO 2	Fatty acid methyl esters of rapeseed oil, surfactants, pH buffering sprays, and antidrifting substance	1.5 l
MSO 3	Surfactants, chelating substance, humectant, and pH buffer	1.5 l
MSO 4	Fatty acid esters of rapeseed oil, surfactants, and pH buffer	1.5 l
NIS	Ethoxylate isodecyl alcohol	0.01%

**Table 6:** Tested and standard adjuvants applied in the field experiments in 2017–2019.

Date of treatment					1 June 2017	24 May 2018	29 May 2019
Temperature (°C)					15.2	17.2	12.8
Relative humidity (%)					70	60	65
Precipitation (mm)					0.0	0.0	0.0
Wind speed (m/s)					0.0	0.0	2.8
Precipitation sum 1–7 days before treatment (mm)					14.0	11.0	19.8
Precipitation sum 1–7 days after treatment (mm)					44.3	0.0	0.1
Temperature during the first week after treatment in 2017							
Date	01/06	02/06	03/06	04/06	05/06	06/06	07/06
Average	19.8	22.7	22.5	19.3	21.4	23.2	18.3
Minimum	9.7	8.1	8.2	14.4	10.3	14.0	12.1
Temperature during the first week after treatment in 2018							
Date	24/05	25/05	26/05	27/05	28/05	29/05	30/05
Average	23.9	25.5	26.7	27.4	29.3	28.7	28.6
Minimum	10.6	11.4	11.6	14.3	14.7	15.1	14.7
Temperature during the first week after treatment in 2019							
Date	29/05	30/05	31/05	01/06	02/06	03/06	04/06
Average	11.9	12.6	16.9	20.1	20.8	22.3	22.7
Minimum	5.4	2.0	7.5	11.7	12.3	11.4	12.1

## Data Availability

Available upon reasonable request.
